# Evaluating the effects of an exercise program (*Staying UpRight*) for older adults in long-term care on rates of falls: study protocol for a randomised controlled trial

**DOI:** 10.1186/s13063-019-3949-4

**Published:** 2020-01-08

**Authors:** Lynne Taylor, John Parsons, Denise Taylor, Elizabeth Binns, Sue Lord, Richard Edlin, Lynn Rochester, Silvia Del Din, Jochen Klenk, Christopher Buckley, Alana Cavadino, Simon A. Moyes, Ngaire Kerse

**Affiliations:** 1 The University of Auckland, Faculty of Medical and Health Sciences, Auckland, New Zealand; 2 Auckland University of Technology, Health and Rehabilitation Research Institute, Auckland, New Zealand; 3 Institute of Neuroscience/Newcastle University Institute for Ageing, Clinical Ageing Research Unit, Campus for Ageing and Vitality, Newcastle University, Newcastle upon Tyne, UK; 4 Newcastle upon Tyne Hospitals NHS Foundation Trust, Newcastle upon Tyne, UK; 5 Institute of Epidemiology and Medical Biometry Ulm, Ulm University, Ulm, Germany; 6 Department of Geriatrics and Geriatric Rehabilitation, Robert-Bosch-Hospital, Stuttgart, Germany; 7 IB University of Applied Sciences Berlin, Study Center Stuttgart, Stuttgart, Germany

**Keywords:** Falls, Exercise therapy, Randomised trials, Aged care, Long-term care, Nursing home

## Abstract

**Background:**

Falls are two to four times more frequent amongst older adults living in long-term care (LTC) than community-dwelling older adults and have deleterious consequences. It is hypothesised that a progressive exercise program targeting balance and strength will reduce fall rates when compared to a seated exercise program and do so cost effectively.

**Methods/design:**

This is a single blind, parallel-group, randomised controlled trial with blinded assessment of outcome and intention-to-treat analysis. LTC residents (age ≥ 65 years) will be recruited from LTC facilities in New Zealand. Participants (*n* = 528 total, with a 1:1 allocation ratio) will be randomly assigned to either a novel exercise program *(Staying UpRight)*, comprising strength and balance exercises designed specifically for LTC and acceptable to people with dementia (intervention group), or a seated exercise program (control group). The intervention and control group classes will be delivered for 1 h twice weekly over 1 year. The primary outcome is rate of falls (per 1000 person years) within the intervention period.

Secondary outcomes will be risk of falling (the proportion of fallers per group), fall rate relative to activity exposure, hospitalisation for fall-related injury, change in gait variability, volume and patterns of ambulatory activity and change in physical performance assessed at baseline and after 6 and 12 months. Cost-effectiveness will be examined using intervention and health service costs.

The trial commenced recruitment on 30 November 2018.

**Discussion:**

This study evaluates the efficacy and cost-effectiveness of a progressive strength and balance exercise program for aged care residents to reduce falls. The outcomes will aid development of evidenced-based exercise programmes for this vulnerable population.

**Trial registration:**

Australian New Zealand Clinical Trials Registry ACTRN12618001827224. Registered on 9 November 2018. Universal trial number U1111-1217-7148.

## Background

Worldwide, falls account for 0.85–1.5% of total annual healthcare expenditures (0.2% of global gross domestic product), with each fall costing up to US$26,000 [1]. Falls are two to four times more frequent in long-term care (LTC) dwellers than in community dwellers [2, 3]. More than 60% of LTC residents fall annually, and fall rates are highest in low dependency care [4, 5], with 5% of LTC residents sustaining a fracture yearly [6].

The mechanisms involved in falls are complex and differ depending on the environmental context and intrinsic risk factors [7]. Video observations of falls in older adults under LTC show two main causes: incorrect weight-shifting during transfers or when turning, and tripping due to inadequate foot clearance [8]. One third of falls in LTC dwellers involve head impact [9], and these falls account for 19% of all traumatic brain injury hospitalisations in older adults [10]. Falls in LTC residents involving head impact most often occur when falling forward whilst walking, with the outstretched hand no longer an effective protective response against impact [9].

Risk factors for falls in LTC occupants are multifactorial. Balance, muscle strength, gait and cognitive impairments all significantly increase the risk of falls [11–13]. Cognitive impairment compromises ‘top down’ control of gait and balance, which is required for navigating the environment [14, 15]. People with dementia are eight times more likely to fall than those without dementia [16], and falls account for 26% of hospital admissions in those with dementia [17]. Increased fall risk in LTC dwellers is associated with a history of falls, walking aid use, moderate disability, wandering, Parkinson’s disease, dizziness, use of sedatives, antipsychotics or antidepressants and higher number of medications used [18].

Management of fall risk reflects this complex presentation, but with mixed outcomes. Pooled results from multifactorial programmes targeting LTC residents’ individual risk profiles show no significant reduction in fall rates [19]. Similarly, pooled results indicate the effect of exercise as a single intervention on the rate of falls in LTC dwellers is uncertain; heterogeneity, protocol weaknesses and small sample sizes limit the conclusions (rate ratio [RaR] 0.93, 95% confidence interval [CI] 0.72–1.20; 2002 participants, 10 studies). Notably, few exercise programmes reviewed were longer than 14 weeks, and the degree of inclusion of people with dementia was not consistently reported. Given that more than 60% of LTC residents have dementia [20], their inclusion in fall prevention programmes is critical. A review of exercise for falls in people with dementia showed positive results [21] (RaR 0.68, 95% CI 0.51–0.91; 781 participants, 7 studies), although few trials were set in LTC facilities.

An exercise program developed by this group, *Staying UpRight*, was successfully piloted in care homes in 2008 [22]. The *Staying UpRight* program is based on an understanding of the physiological systems of balance and includes exercises to challenge these systems, i.e. muscle strength, visual integration, vestibular adaptation, balance strategy retraining and sensory integration. It adheres to principles of rehabilitation, namely appropriate tailoring of dose, intensity and progression by the therapist. Adherence to these principles makes *Staying UpRight* unusual in the LTC setting, where activities are usually undertaken seated, are not tailored to individual ability and are not progressed. The pilot reported improvements in physical function (nonsignificant), with no adverse events and, importantly, attendance of 60% at classes. The program was also acceptable to participants and staff. Based on the success of the pilot, the current trial is powered to test the effect of the *Staying UpRight* program on reducing the rate of falls over a 12-month period.

A second, novel feature of this study is reporting of activity-adjusted fall risk, using wearable accelerometers to measure continuous activity. Falls may reduce as people become less active (reduced exposure to risk), not because of improvement in motor function. An exercise program that improves mobility may therefore increase the number of falls. This potential trade-off has not been tested to date in LTC settings [23–25]. Activity patterns may also provide more insight into the dynamic nature of fall risk [7] and will be explored alongside falls in the context of ongoing cognitive decline.

This study tests the primary hypothesis that the *Staying UpRight* exercise program, when compared to seated exercises, will be effective in reducing falls in LTC facilities. Secondary hypotheses are that the *Staying UpRight* program will:

1. Reduce falls, fall-related injuries and the risk of falling
2. Increase the volume and change the pattern of ambulatory activity
3. Be cost-effective when compared to seated exercise.

## Methods

### Design and setting

**Fig. 1:**
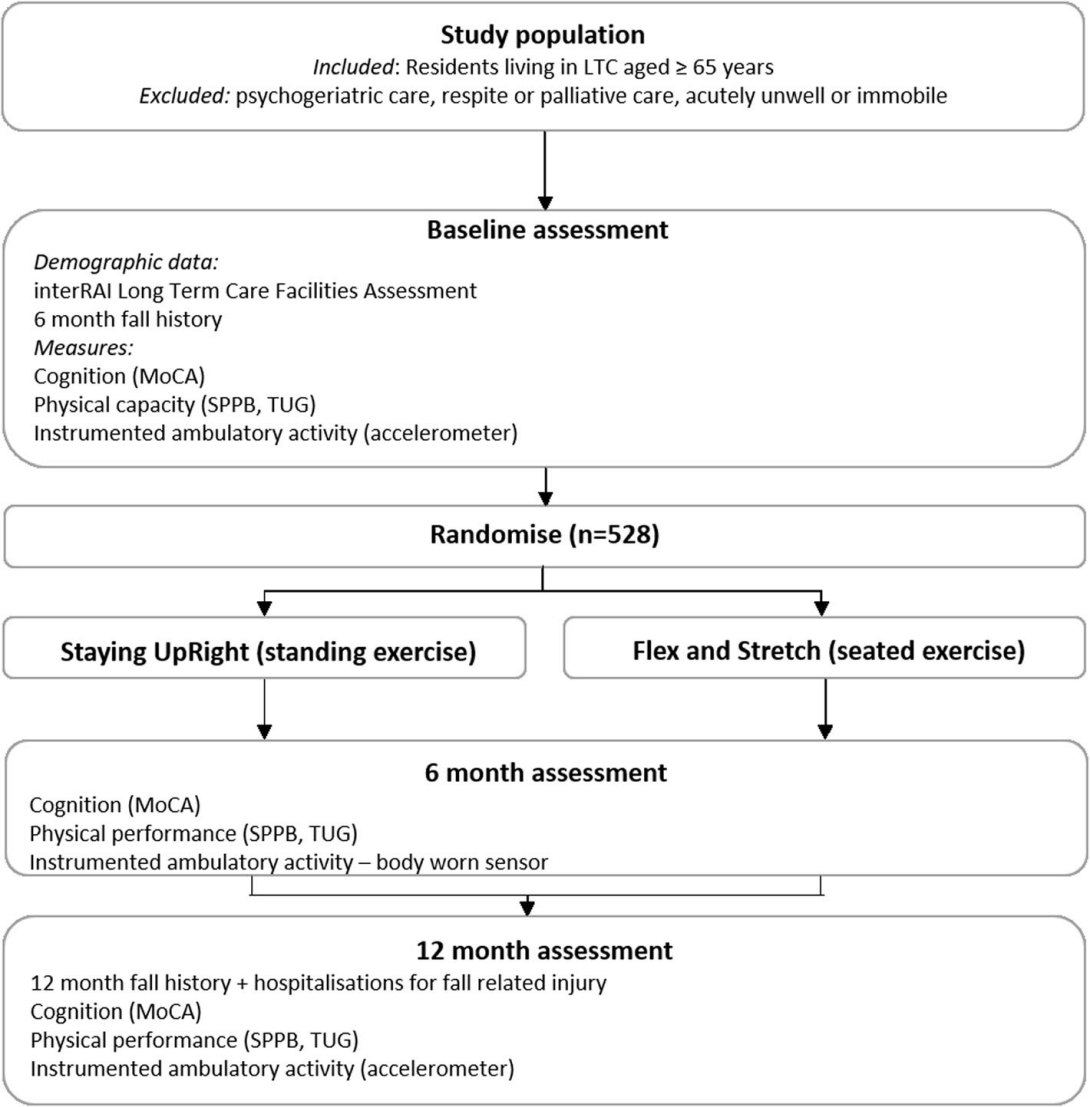
Visual presentation of the study design, including sample, assessments and interventions Abbreviations: *LTC* long-term care, *MoCA* Montreal Cognitive Assessment, *SPPB* Short Physical Performance Battery, *TUG* Timed Up and Go

An investigator and assessor-blinded, parallel-group multisite randomised controlled trial (RCT) will compare a progressive balance and strengthening exercise program (*Staying UpRight*) with a low-intensity seated exercise program (*Flex and Stretch*) provided to older people living in LTC facilities located in Auckland and Hamilton, New Zealand. The study design is outlined in Fig. 1. Reporting of results will conform to the recommendations of the Consolidated Standards of Reporting Trials (CONSORT) statement [26] and to the Standard Protocol Items: Recommendations for Interventional Trials (SPIRIT) guidelines. The SPIRIT checklist is provided as Additional file 2. Any protocol amendments will be submitted to the New Zealand (NZ) Health and Disability Ethics Committee (HDEC) for review and will be updated on the trial registry.

### Participants

Residents aged 65 years or older within participating LTC facilities will be invited to take part. Residents who are in psychogeriatric care, respite or palliative care, acutely unwell or immobile (bed-bound) will be excluded. Study investigators will work with the LTC clinical lead to identify eligible residents. Participants will provide consent before enrolment. For residents unable to provide informed consent due to cognitive impairment, written consent will be sought from the LTC clinical manager, in accordance with NZ HDEC requirements when undertaking research amongst people with cognitive impairment.

### Randomisation

To control for LTC facility factors, randomisation will be stratified by facility and by level of care (high dependency, low dependency and dementia-level care). Levels of care in New Zealand are determined by health authority-appointed Needs Assessment Service Coordination (NASC) agencies. After baseline assessment, participants will be individually randomised to intervention (*Staying UpRight*) or control (*Flex and Stretch*) by a researcher distant from recruitment using a computer-generated random sequence.

### Outcome measures

#### Primary outcome

The primary outcome measure is fall rate (per 1000 person years).

Using the LTC facility’s incident report records, fall registers will be audited for the 6 months prior to study commencement (to provide baseline data) and for the 12 months of the study. Estimates of complete accuracy in fall ascertainment is impossible, as most falls are unwitnessed [4], but the possibility of increased falls being reported as programmes are implemented [27] will be avoided by using the already accepted and implemented reporting systems RiskMan™ (RiskMan International Pty Ltd., Southbank, Victoria, Australia) or VCare™ (VCare International Ltd., Burnside, Christchurch, NZ) that are standardised throughout all LTC facilities in the trial.

#### Secondary outcomes

Secondary outcome measures are:

1. *Risk of falling*, expressed as proportion of fallers per group, i.e. those who sustained at least one fall during follow-up.
2. *Rates of hospitalisation for fall-related injury* (fracture, intracranial or extracranial haemorrhage), expressed as number of fall hospitalisations per 1000 person years of follow-up. Hospitalisation data will be collected from National Health Index (NHI) matched Ministry of Health data over 12 months.
3. *Fall rate relative to activity exposure,* expressed as number of falls per 100,000 steps. Participants will wear a tri-axial accelerometer (Axivity AX3; Axivity, York, UK), secured on the lower back at the fifth lumbar vertebrae (L5) using a hydrogel adhesive (PALStickies, PAL Technologies, Glasgow, UK), covered with an adhesive dressing (OPSITE Flexifix™ and Hypafix™, Smith+Nephew Ltd., Watford, UK). The accelerometer is programmed to sample at a frequency of 100 Hz (range ± 8 g).
4. *Gait volume, pattern and variability of ambulatory activity*, measured using accelerometry as described above. Algorithms are valid for gait spatio-temporal features for macro gait, i.e. volume (total steps, time spent walking, bout number), pattern of activity (bout length and bout distribution), variability of ambulatory bouts and microgait, i.e. pace, rhythm, variability, asymmetry and postural control domains of gait [28–30].
5. *Physical performance*, measured using the Short Physical Performance Battery (SPPB) [31] and the Timed Up and Go (TUG) test [32, 33]. The SPPB comprises ability to stand for 10 s (with feet side by side, semi-tandem and tandem stance), timed chair stand (five times chair rise) and gait speed (measured over 3 m). The maximum score is 12, with higher scores indicating better function. The TUG test requires the participant to rise from a chair, walk 3 m quickly but safely to a mark on the floor, turn, walk back and sit down. A lower time indicates better function.
6. *Cost-effectiveness* of *Staying UpRight* versus seated exercise will be estimated as both an additional cost per fall prevented and per fall requiring hospitalisation.

### Assessments

Demographic and health information (health conditions, medications, independence in activities of daily living) will be collected using a standardised Minimum Data Set (MDS 2.0; interRAI Corporation 1999) at baseline prior to randomisation. The interRAI™ (International Resident Assessment Instrument) Long-Term Care Facilities (LTCF) assessment is a standardised comprehensive observational assessment, completed on admission and 6-monthly thereafter by nursing staff for all residents in LTC.

Cognitive function will be measured at baseline and at 6 and 12 months using the Montreal Cognitive Assessment (MoCA) [34]. The MoCA comprises 16 items to assess multiple cognitive domains. A score of ≤ 10/30 is considered severe cognitive impairment, 11–18 moderate impairment, 19–23 mild and > 23/30 normal cognitive function [35].

Ambulatory activity over 7 days will be collected using the tri-axial accelerometer and data uploaded to an encrypted, secure platform (eScience Central online platform, Newcastle University, UK) [36] for storage and blinded processing.

**Fig. 2:**
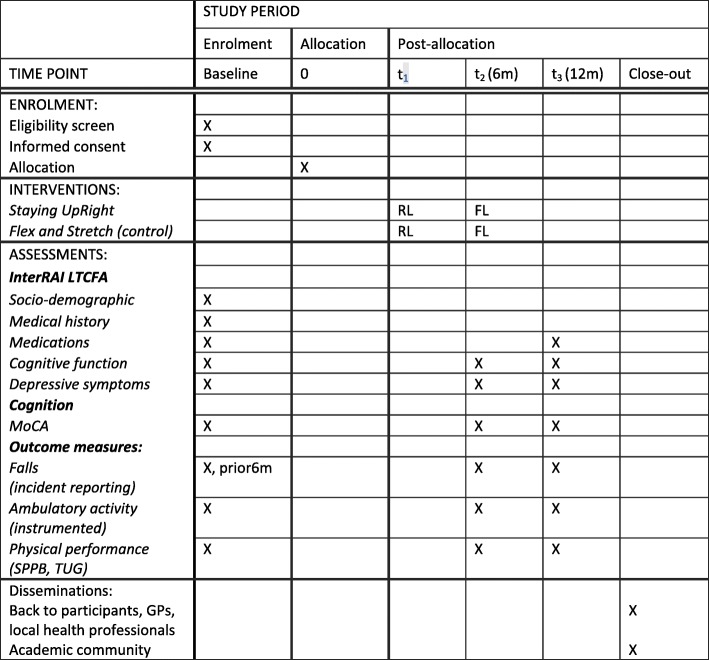
Assessment schedule. *FL* facility lead, *RL* research lead, *InterRAI LTCFA* Long-Term Care Facilities Assessment, *MoCA* Montreal Cognitive Assessment, *SPPB* Short Physical Performance Battery, *TUG* Timed Up and Go

Outcome measures will be completed at baseline and at 6 and 12 months (Fig. 2). Assessors will be trained in all aspects of participant assessment, activity monitoring and digital data management to ensure the assessments are standardised. Assessors will watch a standard assessment and each will rate the responses. Responses will be compared and discussed.

### Interventions

Both intervention and control group participants will participate in any usual activities provided in the LTC facility.

### Blinding

Assessors will be blinded to group allocation for all assessments. Group assignment will only be available to the project coordinator (LT), data manager (SM) and the intervention coordinator (EB) and will be accessed online via a password-protected site. Participants and the staff providing the intervention cannot be blinded.

#### Intervention group

Participants randomised to the intervention group will attend the *Staying UpRight* program. *Staying UpRight* is a supervised balance and strength group exercise program (up to eight per group) delivered for 1 h twice weekly over 1 year. Classes will be led by a physiotherapist trained in programme delivery, supported by an assistant. Balance exercises comprise static and dynamic activities progressed by reducing hand support, reducing the base of support, reducing visual input or adding a cognitive task. The strength exercises use body weight resistance and low repetitions (2 × 10 repetitions at 5–7/10 effort) in weight-bearing positions where possible (Table 1). Exercises are progressed by increasing the number of sets, the speed or amplitude of the movement or the complexity of the task (Table 2). The programme is manualised, with exercises and progressions selected based on participants’ abilities. Exercises completed in each session and exercise progressions for the group will be recorded on a spreadsheet and reviewed as part of fidelity monitoring.

**Table 1:** *Staying UpRight* exercises and progression principles

STRENGTH EXERCISES	
*Progression principles*	
Increase duration of static hold; increase repetition; increase number of sets; alter speed, distance moved, reduce hand support, move from sit to stand, decrease base of support (BoS)	
*Lower limb*	*Description*
Hip extension; abduction, flexion	- Stand up straight, engage core. Lower leg to floor after each movement
Lunges	- Step forwards or sideways. Bend both knees and sink towards floor. Push up with both legs to return. Repeat with alternate leg initiating step
Squats	- Standing behind chair, feet shoulder width apart, bend both knees and sink to the floor. Push up into standing. Progress by reducing hand support, increasing depth of squat, increasing speed
Sit to stand	- Standing up from chair. Progress by reducing hand support, increasing speed
Heel raises and toe raises	- Standing behind chair, lifting heels off the floor. Lifting toes off the floor.
*Upper limb*	
Breaststroke	- Hands together in front of chest. Push arms forward, pull to side & back as if doing breaststroke
Shoulder press	- Hands at shoulder level, elevating arms to ceiling through flexion. Progress from seated to standing, increase speed
Biceps curl	- Elbow flexion
Triceps chair press up	- Hands on seat of chair, back straight. Push down through arms to lift bottom off seat. Slowly bend elbows and lower
Wall press	- Stand with hands on wall at shoulder height. Keep back & legs straight, bend elbows and lower body towards the wall. Straighten elbows and return to standing
Boxing	- Jabs: make a fist punch forward alternating left & right. Hooks: make a fist and punch across your body
	- Uppercuts: Make a fist and punch upwards from waist to chin. Progress from seated to standing, increase speed
*Trunk*	
Trunk flexion, extension, side flexion, rotation	- Progress from seated to standing
BALANCE EXERCISES	
*Progression principles: static exercises*	
- Increase duration of static hold	
*Progression principles: dynamic exercises*	
- Increase or vary speed; require stop, start or change direction on command	
*Progression principles: all exercises*	
- Reduce hand support, BoS, visual input; add a cognitive task; combine exercises & progressions	
*Centre of gravity*	***Description***
Low level	- Stand with chair support both hands, one hand
	- Stand unsupported, arms by side
	- Stand arms crossed
	- Feet together; semi-tandem stance; tandem
	- Arms to side; arms crossed
Medium level I	- Reduce BoS: feet together, move to semi-tandem stand
	- Reduce sensory input: eyes closed
	- Add cognitive task: count backwards out loud
High level II	- Reduce BoS and add arm movements: unilateral to bilateral
	- Standing + throw & catch: reduce BoS + throw & catch
*Weight transfer within limits of stability*	
Low level	- Standing shift weight foot to foot
	- Standing move hips in circular figure of 8 patterns
	- Marching
Medium level II	- Nudge object on ground in different directions with foot; pass between feet and to neighbour
High level I	- Reduce BoS (feet together, semi-tandem, tandem) reach: turn & reach
	- Pass ball/object between group members; vary height & distance of reach
	- Alternating top taps forwards, backwards, sideways, diagonally
*Postural strategy: challenging limits of stability*	
Low level	- Standing sway: forwards & return to midpoint. Repeat backwards/lateral directions
	- Standing pass object (newspaper, scarf) around body
Medium level	- Standing sway forwards, backwards, sideways, diagonal *without stopping at midpoint.* Increase sway speed/distance
	- Squat and pass object between legs in figure of 8 pattern
High level	- Standing sway eyes closed
	- Step forwards, backwards, sideways, lean & step; increase speed and step length
GAIT TRAINING	
Low level	- Step forwards, backwards, sideways, diagonally
	- Walk on spot/on toes/heels
	- Turn clockwise/anticlockwise around chair
High level II	- Tandem walk forwards/backwards
	- Step over obstacles
MULTISENSORY TRAINING	
Low level (sitting)	- Fix eyes on finger: follow moving finger keep head still
	- Fix eyes on point ahead, move head slowly side to side, up & down, diagonally
	- Fix eyes on point ahead, move head slowly side to side, up & down, diagonally
	- Sit unsupported with eyes closed
Medium level II (standing)	- Stand still, eyes closed
	- Turn trunk in same direction as turning head & pass object to neighbour turning trunk in same direction as head. Swing arms/scarf with trunk & head whilst watching arms/scarf
High level	- Reduced BoS. Fix eyes on finger & track moving finger, head still
	- Reduced BoS. Fix eyes on point & move head slowly side to side, up & down, diagonally keeping eyes fixed
	- Reduced BoS, eyes closed

*Note:* The table shows selected exercises. The full programme is available on request from the corresponding author

**Table 2:** *Staying UpRight* class structure

Weeks 1–2	5 min	Breathing control/posture
Balance	10 min	1–2 exercises from each category^a^
Strength	20 min	10 repetitions × 2 sets; 60 s rest
		Upper limb × 2–3
		Trunk × 2
		Lower limb × 3–4
Cool down	5 min	Stretching
Weeks 3–4	5 min	Breathing control/posture
Balance	10 min	1–2 exercises from each category^a^
Strength	20 min	15 repetitions × 2 sets; 60 s rest
		Upper limb × 2–3
		Trunk × 2
		Lower limb × 3–4
Cool down	5 min	Stretching
Weeks 5–6	5 min	Breathing control/posture
Balance	15 min	2–3 exercises from each category^a^ (introduce new exercises & progress previous)
Strength	25 min	12–15 repetitions × 2 sets; 60 s rest
		Upper limb × 3
		Trunk × 2–3
		Lower limb × 4
Cool down	5 min	Stretching
Weeks 7–13	5 min	Breathing control/posture
Balance	15 min	3–4 exercises from each category^a^ (Introduce new exercises & progress previous)
Strength	30 min	15 repetitions × 2 sets; 60 s rest
		Upper limb × 3–4
		Trunk × 2–3
		Lower limb × 5–6
Cool down	5 min	Stretching
Week 14–ongoing	5 min	Breathing control/posture
Balance	20 min	3–4 exercises from each category^a^ (introduce new exercises & progress previous)
Strength	30 min	15 repetitions × 2 sets; 60 s rest
		Upper limb × 4
		Trunk × 3
		Lower limb × 6
Cool down	5 min	Stretching

^a^Balance categories: centre of gravity control, weight transfer, postural strategies, gait training, multisensory

To test sustainability, the LTC facility staff (physiotherapist or physiotherapy assistant) will take over delivery of the classes for the second 6 months, ensuring continuity of the programme for 1 h twice weekly over the 12-month period. If the ongoing provision of classes is compromised within a facility, alternative funding streams will be sought.

#### Control group

Control group participants will attend a seated group activity programme (*Flex and Stretch*) delivered for 1 h twice weekly over 1 year. Classes are led by the LTC activities staff or a volunteer trained in programme delivery. Activities comprise lower limb, trunk, upper limb, head and neck movements without resistance or progressions, e.g. seated swimming, boxing, seated marching, heel and toe tapping and seated stretches; as well as activities, e.g. balloon catch and throw, pass the parcel. The programme is manualised, with class duration for each session recorded.

#### Class attendance

Class attendance, class duration and reasons for nonattendance will be documented for both intervention and control groups.

#### Intervention fidelity monitoring

To ensure fidelity of the intervention, an exercise class at each facility will be observed by a research investigator within the first 2 months of physiotherapist delivery and within the second 6 months of LTC facility delivery. A fidelity checklist which includes the number of participants, exercises completed, total time spent in standing and total class duration will be used to identify any deviations from the exercise protocol, with feedback given to the class facilitator. The research intervention coordinator will also audit all class exercise spreadsheets monthly to identify any deviations from the protocol.

### Contamination

Contamination between intervention and control groups is best controlled by a cluster randomised design, but that also introduces considerable heterogeneity given differences in length of stay, spatial design of the LTC and differences in staffing ratios. Fall rates in facilities in a previous trial varied from 0.68 fall per resident year to 7.67 falls per resident year [37]. LTC facility factors found to be associated with falls included the level of care (low-level dependency had the highest rate of falls) and staffing ratios [5]. Individualised randomisation addresses these issues, although it introduces the possibility of contamination. Contamination between intervention and control groups will be managed by separating delivery of the intervention and control classes, maintaining attendance registers for intervention and control classes and using different facilitators for intervention and control groups.

### Risk management and safety monitoring

In the event of a fall or medical event, standard LTC facility procedures will be followed. Falls, mortality and unplanned re-admissions to an acute hospital service sustained during the trial period will be reported to an independent Data Monitoring Committee (DMC; see project governance) and reported to the relevant ethics committee.

### Data management

All data will be stored in a confidential manner. Participants will be assigned a code which will be used for all data management and analyses. Paper data will be stored at the local site in a locked filing cabinet. Coded electronic data will be stored on a computer server at the host organisation (the University of Auckland) for the duration of the study. Access to the computer files will be password-protected and accessible only to the research team. Data quality will be monitored by the data manager on a regular basis and reported to the DMC. All project investigators will have access to the final, de-identified data set.

### Sample size estimation

The sample size is estimated on the primary outcome, fall rate. To detect a 25% reduction in falls, assuming a control rate of 2.6 falls per resident per year (based on pilot data), we estimate a required sample size of 264 in each group (*n* = 528; two-tailed test, a = 0.05, power = 90%). The anticipated drop-out rate is 35%, which will be replaced by recruitment at participating facilities throughout the trial. Final recruitment will stop 12 weeks prior to completion of the 12-month intervention in each facility.

### Statistical analysis

The primary analysis will be conducted on an intention-to-treat basis using data from all randomised participants, although a ‘per protocol’ analysis of the primary outcome will also be reported.

Total number of falls, number of fallers, people sustaining a fall-related fracture or brain injury; fall rate (falls per 1000 person years); multiple fallers and number in each analysis will be reported. Prior fall-incidence rates will be calculated as number of falls/resident/year using the audited 6 months prior to enrolment.

Negative binomial regression models will be fitted to determine the incidence rate ratio (IRR) for differences in fall rates between groups both for overall follow-up time as the primary endpoint and secondarily as activity-adjusted rates. Similar negative binomial models will be built for fall injury rates; fractures and head injuries both combined and separately. Logistic regression models for fallen during follow-up or not fallen and for having fallen multiple times during follow-up or not fallen will be compared between the intervention and control groups.

All models will control for prior fall rate, level of dependency and cognition (MoCA), as these are confounding factors. Baseline data will be compared between the two groups, with any strongly imbalanced factors further adjusted for in the analysis.

Per protocol analyses will be performed including those with higher attendance, and pre-planned subgroup analyses will include those with moderate and high levels of cognitive impairment.

Fall rate relative to activity exposure will be calculated and compared between groups. Within-group change will be examined using repeated measures. Generalised linear mixed-effects regression models will be used for volume, pattern and variability of ambulatory activity and cognition.

The cost-effectiveness model will look at the difference in total costs of hospitalisation due to fracture or head injury and overall between intervention and control groups, compared to the difference in the cost of the two exercise programmes. Unit costs will be assigned using Weighted Inlier Equivalent Separation New Zealand (WIESNZ) by type of hospitalisation, indicative District Health Board costs for outpatients and literature/expert judgement for anything not already covered. Incremental cost-effectiveness for intervention versus comparator activity will be assessed (1) per fall prevented and (2) per injury fall (resulting in hospitalisation) prevented. The cost-effectiveness of *Staying UpRight* as an adjunct to usual activity will be explored for these same outcomes by removing the costs of the comparator activity (retaining any effect). Sensitivity analyses will be performed for all analyses to indicate the uncertainty in our estimation of the costs and consequences using bootstrapping. Decision uncertainty will be assessed by means of a probabilistic sensitivity analysis using standard diagrams (cost-effectiveness acceptability curve/cost-effectiveness frontier) and varying assumptions around LTC facility size.

A range of further sensitivity analyses will be considered, including modifying the potential size of attendance within the intervention or control groups (where not fully subscribed), fall-related versus all-cause hospitalisation costs as well as the likely cost of other healthcare utilisation in LTC.

### Project governance

The Principal Investigator (PI) is responsible for overseeing all aspects of the project. The University of Auckland’s research processes oversee the trial and provide financial integrity.

Ongoing monitoring of the study for futility, data integrity and safety will be conducted by the external independent DMC. The DMC will meet every 6 months. One of these meetings will involve formal review of interim statistical analyses. A nominated member of the DMC will be provided immediate access on an ongoing basis to patient-specific information on suspected unexpected serious adverse reactions.

Internal monitoring is through the Steering Committee and the Operations Committee (PI and key researchers), who are responsible for project management including recruitment, assessment and intervention delivery.

### Dissemination policy

The results of the trial will be submitted to international peer-reviewed journals and presented at conferences. Decisions about publications arising from the data set will be ratified by the Steering Committee, and all named investigators will be eligible for authorship. Trial staff will also provide workshops to clinicians to assist in the translation of findings to clinical practice.

## Discussion

Older people fall frequently in LTC facilities with disastrous consequences, including injury and hospitalisation. Preventing falls in these LTC residents has been difficult. This study tests a sustained balance and strength exercise programme designed for LTC residents, including those with dementia, compared to a seated exercise programme to determine if falls and injury from falls can be prevented.

A novel feature of this study is the evaluation of fall rate relative to activity exposure. The relationship between ambulatory activity levels and fall risk exposure has not been tested in LTC settings. Understanding the relationships between ambulatory activity and risk of falls in individuals will lead to a more personalised approach to fall prevention in LTC settings.

Cost-effectiveness analyses will determine whether there is a return on investment. If this exercise programme is successful, those in LTC will benefit and costs may be reduced.

### Trial status

The trial commenced recruitment on 30 November 2018 and is currently open for recruitment. The recruitment target is 528 participants. It is anticipated that this target will be reached by June 2020. The protocol is version 7, dated August 22, 2019.

### Hardware

The AX3 data logger is provided by Axivity Ltd., The Core, Bath Lane, Newcastle Helix, Newcastle upon Tyne, NE4 5TF, UK.

### Incident reporting software

The RiskMan software is a product of RiskMan International Pty Ltd., 11 Meaden Street, Southbank, Victoria, Australia 3006. The VCare software is provided by VCare International Ltd., 7/35 Sir William Pickering Dr., Burnside, Christchurch 8053, New Zealand. The International Resident Assessment Instrument (interRAI) is provided by interRAI™ NZ, 69 Tory Street, Wellington 6140, New Zealand.

## Supplementary information

**Additional file 1:** Ethics consent and information forms.

**Additional file 2:** SPIRIT 2013 checklist.

## Data Availability

Not applicable.

## Abbreviations

ACTRN: Australian New Zealand Clinical Trials Registry registration number
CEAC: Cost-effectiveness acceptability curve
CEF: Cost-effectiveness frontier
CONSORT: Consolidated Standards of Reporting Trials
HRC: Health Research Council of New Zealand
interRAI: International Resident Assessment Instrument
LTC: Long-term care
MoCA: Montreal Cognitive Assessment
PI: Principal Investigator
QALY: Quality-adjusted life year
RCT: Randomised controlled trial
SPPB: Short Physical Performance Battery
TUG: Timed Up and Go
WIESNZ: Weighted Inlier Equivalent Separation New Zealand
