# Supplementary material for: Evaluating the effects of an exercise program (Staying UpRight) for older adults in long-term care on rates of falls: study protocol for a randomised controlled trial
Source: Trials. 2020 Jan 8;21:46. doi: 10.1186/s13063-019-3949-4 (PMC6950827; doi:10.1186/s13063-019-3949-4)
Supplement: Supplementary file 1 — Additional file 1: Ethics consent and information forms. [file 13063_2019_3949_MOESM1_ESM.docx]

*Available in English*

**1. Consent Form for Participants:**

**Study Title: Staying Upright in Residential Care**

| I have read or have had read to me and I understand the Participant Information Sheet. |  | 🞏 |
| --- | --- | --- |
| I have been given enough time to consider whether or not to participate in this study. |  | 🞏 |
| I have had the opportunity to use a legal representative, whānau/ family support or a friend to help me ask questions and understand the study. |  | 🞏 |
| I am satisfied with the answers I have been given regarding the study and I have a copy of this form and information sheet. |  | 🞏 |
| I understand that taking part in this study is voluntary and that I may withdraw from the study at any time without this affecting any medical care. |  | 🞏 |
| I consent to the research staff collecting and processing information, including information about my health. |  | 🞏 |
| If I decide to withdraw from the study, the information collected about me up to the point of withdrawal may continue to be processed. |  | 🞏 |
| I understand that participation in this study is confidential and that no material, which could identify me personally, will be used in any reports on this study. |  | 🞏 |
| I know who to contact if I have any questions about the study in general. |  | 🞏 |
| I understand the responsibilities of study participation. |  | 🞏 |

**Please tick to indicate you consent to the following**

| I consent to participate in the study | Yes 🞏 | No 🞏 |
| --- | --- | --- |
| I wish to receive a summary of the results from the study. | Yes 🞏 | No 🞏 |
| I consent to the research team having access to care home, hospital and interRAI records. | Yes 🞏 | No 🞏 |
|  |  |  |

**Declaration by participant**

I hereby consent to participate in this study.

| Participant’s name: | |
| --- | --- |
| Signature: | Date: |

**Declaration by researcher:**

I state that I have fully explained the study and the resident has the capacity to give informed consent.

| Researcher’s name: | |
| --- | --- |
| Signature: | Date: |

**2. Statement of Support for Family/Friends of Participants:**

**Study Title: Staying Upright in Residential Care**

| I have read or have had read to me, and I understand the Participant Information Sheet. |  | 🞏 |
| --- | --- | --- |
| I have been given enough time to consider whether my relative/friend would like to participate in this study. |  | 🞏 |
| I am satisfied with the answers I have been given regarding the study and I have a copy of this form and information sheet. |  | 🞏 |
| I understand that taking part in this study is voluntary and that my relative/friend may withdraw from the study at any time without this affecting any medical care. |  | 🞏 |
| I think that my relative/friend would have consented to the research staff collecting and processing information, including information about the health of my relative friend. |  | 🞏 |
| If my relative/friend decides to withdraw from the study, the information collected about them up to the point of withdrawal may continue to be processed. |  | 🞏 |
| I understand that participation in this study is confidential and that no material, which could identify my relative/friend personally, will be used in any reports on this study. |  | 🞏 |
| I know who to contact if I have any questions about the study in general. |  | 🞏 |
| I understand the responsibilities of study participation. |  | 🞏 |

**Please tick to indicate your support of the following**

| I think my relative/friend would have consented to doing the exercise classes if they had the capacity to decide. | Yes 🞏 | No 🞏 |
| --- | --- | --- |
| I wish to receive a summary of the results from the study. | Yes 🞏 | No 🞏 |
| I think my relative/friend would have consented to contacting their GP if needed | Yes 🞏 | No 🞏 |
| I think my relative/friend would have consented to the research team having access to care home, hospital and interRAI records | Yes 🞏 | No 🞏 |

**Declaration by family member of participant**:

I hereby state that I think my relative would have consented to take part in this study if they had the capacity to decide.

Participant’s name: __________________________________________

Family/friend’s name_________________________________________

Signature: ________________________________Date: ____________

**Declaration by clinician:**

I consider that participation in this service innovation study about activity and gait and falls is in the best interests of the resident.

Clinician’s name: **_________________________, Clinical Lead**

Signature: ________________________________Date: ____________

**3. Consent Form for Care Centre Management:**

**Study Title: Staying Upright in Residential Care**

| I have read and I understand the Information Sheet. | 🞏 |
| --- | --- |
| I understand that this care home and staff taking part in this study is voluntary and that I may withdraw the care home from the study at any time without this affecting any care to residents. | 🞏 |
| I understand that participation in this study is confidential and that no material, which could identify this care home personally, will be used in any reports on this study. | 🞏 |
| I know who to contact if I have any questions about the study in general. | 🞏 |
| I understand the responsibilities of study participation. | 🞏 |

**Please tick to indicate you give permission to the following**

| I give permission for staff working with researchers to facilitate the study | Yes 🞏 | No 🞏 |
| --- | --- | --- |
| I wish to receive a summary of the results from the study. | Yes 🞏 | No 🞏 |
| I will work to continue the exercise classes for the second six months of the trial | Yes 🞏 | No 🞏 |
| I give permission to the research team having access to care home, hospital and interRAI records with permission of the residents. | Yes 🞏 | No 🞏 |
|  |  |  |

**Declaration by management:**

I give permission for this care home and staff to facilitate this study.

| Care home name:  Manager’s name: | |
| --- | --- |
| Signature: | Date: |

**Declaration by researcher:**

| I state that I have fully explained the study and provided the manager with a copy of the Information Sheet  Researcher’s name: | | |  |
| --- | --- | --- | --- |
| Signature: | Date: |  | |

**4. Participant Information Sheet**

**Study Title:** **Staying Upright in Residential Care**

**Ethics Committee Reference:** **18/NTB/151**

**Locality:** University of Auckland

**Lead Investigator:** Ngaire Kerse

**Contact Number:** XXXXX

|  |  |  |  |
| --- | --- | --- | --- |

Dear resident

My name is Ngaire. I am a GP and work at the University of Auckland.

I would like to invite you to take part in a study about staying strong and upright in residential care. You are invited because you live in a care home. Taking part in this study is your choice. You do not need to give a reason if you do not want take part. There will be no change in your care at the home whether you take part of not. If you take part in the study and change your mind later, you can withdraw from the study at any time.

The other people on this study are: Dr Lynne Taylor, Dr John Parsons, Dr Denise Taylor, Ms Liz Binns, Dr Sue Lord, all physiotherapists interested in care homes and Dr Anna Rolleston, a Māori health and wellness researcher.

This information sheet tells:

- why we are doing the study, what taking part would involve,
- what the benefits and risks might be, and what will happen at the end of the study.

We will talk about this information with you and answer any questions you may have. If you agree to take part in this study, you will be asked to sign the Consent Form. You will be given a copy of both this information sheet and the Consent Form to keep.

This document is 6 pages long. Please read this information sheet. It is important that you understand all the pages.

**What is the purpose of the study?**

People in residential care probably walk a lot less than people living at home. Many will have some memory problems which makes walking harder. Exercise classes may help you stay strong, but we don’t know exactly what type of exercise is best. This study tests exercises to see how they strengthen, improve function and prevent falls. Some participants will do exercises standing up and using some weights. Others will do seated exercises concentrating on flexibility. A facilitator, trained physiotherapist will direct the classes and an additional volunteer may assist. The choice of which exercises you do is made by chance and about half of the participants will do each type of exercises. The classes are an hour long, have warm up and warm down periods and exercises in the middle, will be twice a week and we hope the programme will go on for a year. This study is also very interested in how people walk, and how the style and amount of walking change over time. Some styles of walking mean the risk of falls is higher. We would like to see how the exercises improve the way people walk and prevent falls. The study has been funded by the Health Research Council of New Zealand. The host institution is the University of Auckland and researchers from AUT University, Newcastle University, Stuttgart University are helping. Please contact Ngaire Kerse XXXXXXXXXX if you have any questions.

This study has been approved by the Health and Disabilities Ethics Committee, Reference Number: **18/NTB/151**

**What will my participation in the study involve?**

If you participate, we will offer you an exercise class to attend for an hour twice a week for a year. For the first six months this will be led by a physiotherapist or trained leader funded by the study, and the second six months the care home you live in will organize the classes.


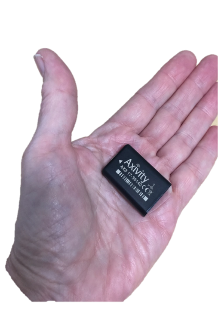
Before we start, we would like to ask you some questions about your memory and ask you to stand up and sit down, test your balance and time you while you are walking. This will take about 20 minutes.

Another way to measure walking is to wear a small device that monitors movement called an accelerometer. This is a picture of the monitor. It is about 2x3 cm and the picture below shows it attached to a man’s back. It is attached to your back with tape. We would like you to wear it for a week to get a good profile of activity.


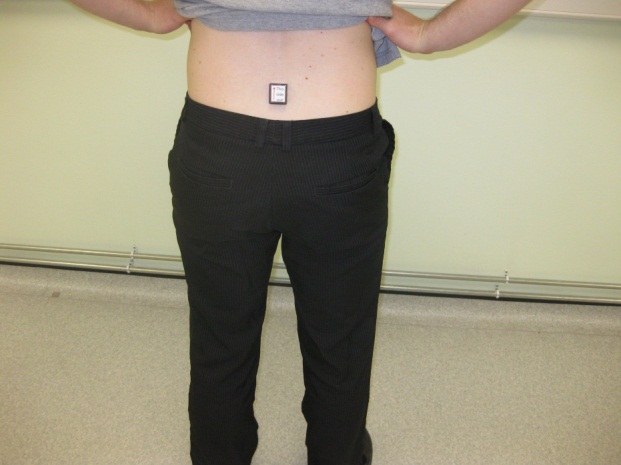
The interview and putting the activity monitoring happens at the beginning, after 6 months and at 12 months which is the end of the study. If there is any skin itching or the monitor is uncomfortable it can be taken off easily.

Please could we have your permission to look at the care home records about you for 18 months before and during the study? This is to save asking you lots of questions. The records include any falls, your medications, the assessments the nurse does every 6 months. Please could we also look at the hospital records and Ministry of Health records about you? This is to work out whether the classes improve health.

If your care facility decides to withdraw from the study, we will work with them to maintain availability of the activity programmes. It is possible these will stop if the care home management decides to withdraw.

**What are the possible benefits and risks of this study?**

We think the risks in taking part in this study are small. The exercise classes have been tried out in residential care and worked well. There is a small chance that you will be tired or will fall during a class. If this happens the leaders of the class will look after you and the nurses and doctors at the care home are nearby. The monitor may be uncomfortable and can easily be taken off.

If there is a significant health or safety issue detected during the study, the staff will discuss this with you and, with your permission, talk to the nurses and doctors at the care home.

**Who pays for the study?**

Taking part in this study will not cost you any money. The classes are free to you and paid for by the research grant.

**What are my rights?**

Taking part in this study is voluntary. You are free to *not* take part in the study and you don’t have to give a reason for doing so. If you do want to take part now, but change your mind later, you can pull out of the study at any time. There will be no change in your care at the care home whether you participate or not.

All the information collected will be kept confidential, your details and any identifying information will be removed, and only the researchers will look at the information collected from and about you. Nothing that could identify you will be used in any reports about this study. Any place or name that could lead to you being identified will be removed. You have the right to access information that has been collected about you as part of the study.

If you were injured in this study, which is unlikely, you would be eligible to apply for compensation from ACC just as you would be if you were injured in an accident at work or at home. This does not mean that your claim will automatically be accepted. You will have to lodge a claim with ACC, which may take some time to assess. If your claim is accepted, you will receive funding to assist in your recovery. If you have private health or life insurance, you may wish to check with your insurer that taking part in this study won’t affect your cover.

**What happens after the study or if I change my mind?**

After the study finishes the care homes will know how to continue the classes at no cost to you if they improve strength and reduce falls. The study data will be stored in a locked cabinet in the University of Auckland throughout the duration of the study and all computer files and e-records about the study are password protected. At the end of the study Ngaire will make sure the data is stored in a secure place in the School of Population Health at the University of Auckland. It will be kept for 10 years and then destroyed.

The study data may be used in future studies, we would like to combine the activity and falls data with other studies in care homes in New Zealand and overseas to better understand how memory loss affects the way people walk and contributes to the risk of falls.

A summary of the study findings will be presented back at a public meeting within 2 years of completion of the study and articles in health journals and books will be written about the study.

**Who do I contact for more information or if I have concerns?**

If you have any questions, concerns or complaints about the study at any stage, you can contact:

Name XXX Phone XXX

If you want to talk to someone who isn’t involved with the study, you can contact an independent health and disability advocate on:

Name XXX Phone XXX

Email: [xxx](mailto:advocacy@hdc.org.nz)

If you wish to talk to a Māori health advisor, you can contact

Name XXX Phone XXX

Email: [xxx](mailto:advocacy@hdc.org.nz)

You can also contact the Health and Disability Ethics Committee (HDEC) that approved this study on:

Phone: 0800 4 ETHICS

Email: XXX
